# Neural network reconstruction of the left atrium using sparse catheter paths

**DOI:** 10.1007/s11548-024-03268-y

**Published:** 2024-09-16

**Authors:** Alon Baram, Moshe Safran, Tomer Noy, Nave Geri, Hayit Greenspan

**Affiliations:** 1 Biosense Webster (Israel), Ltd, 4 Hatnufa Street, 20692 Yokneam, Israel; 2 Medical Image Processing Laboratory, Department of Biomedical Engineering, Faculty of Engineering, Tel Aviv University, 69978 Tel Aviv, Israel; 3 Department of Radiology, Icahn School of Medicine, Mount Sinai, New York, NY USA; 4 RSIP Vision, 16 King George, 94229 Jerusalem, Israel

**Keywords:** Left atria reconstruction, Neural networks, Minimally invasive electrophysiology, Dense encoder decoder, Convolutional neural network

## Abstract

**Purpose:**

Catheter-based radiofrequency ablation for pulmonary vein isolation has become the first line of treatment for atrial fibrillation in recent years. This requires a rather accurate map of the left atrial sub-endocardial surface including the ostia of the pulmonary veins, which requires dense sampling of the surface and currently takes more than 10 min. The focus of this work is to provide left atrial visualization early in the procedure to ease procedure complexity and enable further workflows, such as using catheters that have difficulty sampling the surface.

**Methods:**

We propose a dense encoder–decoder network with a novel regularization term to reconstruct the shape of the left atrium from partial data which is derived from simple catheter maneuvers. To train the network, we acquire a large dataset of 3D atria shapes and generate corresponding catheter trajectories, from which traversed point clouds are obtained. Once trained, we show that the suggested network can sufficiently approximate the atrium shape based on a given trajectory.

**Results:**

We compare several network solutions for the 3D atrium reconstruction. We demonstrate that the solution proposed produces realistic visualization using partial acquisition within a 3-min time interval using human clinical cases.

**Supplementary Information:**

The online version contains supplementary material available at 10.1007/s11548-024-03268-y.

## Introduction

### Clinical problem

**Fig. 1:**
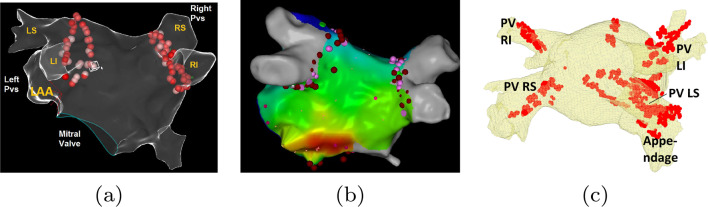
Clinical use of LA surface. **a** LA anatomy and typical ablation (red points), **b** LA electric propagation map. **c** Initial bearing path in red, CT anatomy is in yellow. Most of the red points lie inside the blood pool (not over the surface)

Atrial fibrillation (AF) is the most prevalent form of cardiac arrhythmia, affecting millions of people worldwide each year. It poses a significant health concern due to its association with an increased risk of embolic stroke and decreased quality of life [1]. Currently, the first line of treatment for AF is catheter-based electro-anatomic mapping (EAM) 3D-guided radiofrequency ablation for pulmonary vein isolation (PVI) [2], as shown in Fig. 1a. This approach is rapidly gaining popularity and success as it targets the primary source of AF triggers located in the pulmonary veins.

EAM systems play a crucial role in catheter ablation treatment methods, as they record the position and electrical signals acquired as the catheter is navigated within the heart chamber. These data points are then used to reconstruct the endocardial surface, providing visualization of the left atrial (LA) anatomy, including the anatomical parts of the pulmonary veins (PVs) such as the left superior (LS), right superior (RS), left inferior (LI), right inferior (RI), and left atrial appendage (LAA) [3]. The reconstruction process relies on local activation times to approximate the electrical propagation wave over the LA surface, responsible for the chamber’s contraction, as depicted in Fig. 1b.

Current electrophysiological (EAM) systems have limitations. Achieving accurate anatomically correct LA surfaces requires catheter contact along much of the blood pool and tissue boundary, demanding skilled and time-consuming maneuvers by physicians and manual operator result editing, often taking tens of minutes [4]. Additionally, post-PVI, other arrhythmias can complicate the procedure [2]. Special care is needed for certain regions like the esophagus or deep pulmonary veins [5]. To overcome these challenges, imaging methods like MRI and intra-cardiac ultrasonic catheters can enhance anatomical understanding. However, each system has its own limitations. X-ray and CT face issues with radiation and time, while X-ray and ultrasound are constrained by their field of view. MRI is affected by noise, cost, and availability, and there are also challenges related to changes in heart shape [6]. Enhanced imaging, especially in challenging areas like the PVLS and LAA ridge, can improve catheter navigation and tissue contact visualization during ablation [7].

The current study focuses on efficiently mapping the LA endocardial surface using a portion of the catheter traversal path, which is a common workflow for physicians. For initial bearing, the physician maneuvers the catheter through a path that traverses known anatomical landmarks, such as the four pulmonary veins ostia, and is acquired in under 3 min. First, the catheter is inserted through the trans-septal puncture from the right atrium to the left. It is then pushed until it reaches the left pulmonary veins ostia (either directly or by sliding along the roof). The ostia of either the LS or LI is encountered, and the physician identifies both using clues from the ECG. The catheter is then maneuvered to the right side (along the roof) to identify the veins on the right side. An example of this path, alongside the corresponding anatomical shape, is shown in Fig. 1c. By providing LA surface visualization at an early mapping stage, we aim to reduce mapping time while maintaining anatomical accuracy, particularly in the PV ostia, which are critical landmarks for ablation success. Our system is trained to accommodate LA anatomy variations with four PVs, representing a majority of the population. Note that we assume that for the less common anatomical variations, different models can be trained and engaged by the physician appropriately. The reconstructed PV openings and orientations should exhibit minimal errors, facilitating easy identification of anatomical parts for improved clinical outcomes in AF treatment. By optimizing mapping procedures and integrating anatomical imaging guidance, we can advance the effectiveness and safety of catheter ablation techniques for AF and other related arrhythmias.

### Proposed solution

**Fig. 2:**
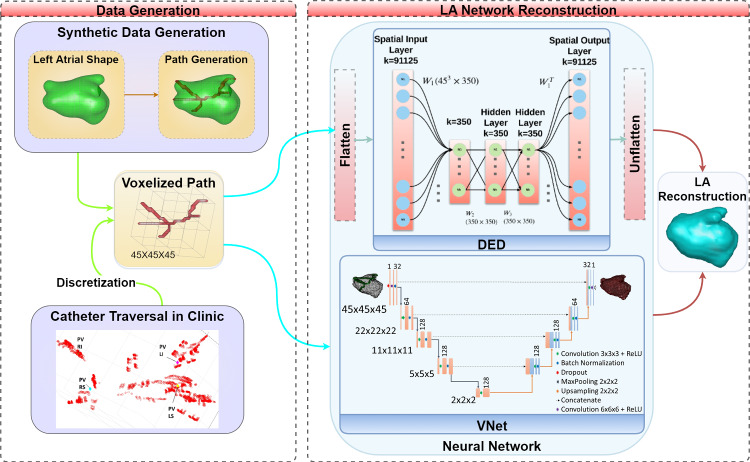
System block diagram: in the data generation step (left), a path is obtained via either synthetic generation or by a clinical acquisition. For generating the training data, the LA shape is obtained from CT or model; then, the synthetic path is generated (in red) and passed to the network; in a clinical setting, a point cloud is sampled by the catheter, registered, and discretized; The network (DEDor V-Net) outputs the most probable atrium (right)

The proposed method involves two main steps: (1) path generation and (2) atria shape reconstruction, as shown in Fig. 2. In the first step, we utilize a statistical model to generate LA shapes. The model is described in “Related work” section. The generation step is necessary due to the limited accessibility of patient data. We employ a novel algorithm to generate catheter paths within the generated shapes. These paths are represented as point clouds (without the temporal component), as is described in “Methods” section. In the second step, we reconstruct LA shapes from catheter inputs using neural networks.

We present a network solution which we term the dense encoder–decoder network (DED) network. We show that this solution provides anatomically relevant outputs and outperforms other baseline methods. We demonstrate time efficiency, allowing for early guidance, as well as strong accuracy in real-world clinical data inputs.

This study builds upon a preliminary conference version [8]. We start in “Related work” section with an overview of relevant literature. In “Methods” section, we define the problem and detail our proposed solution. Experiments and methodological evaluations are presented in “Experiments and results” section. Results include real clinical cases. “Discussion and conclusion” section concludes the work with a discussion of the method and results.

## Related work

EAM systems are now common for treating arrhythmia during electrophysiology procedures. The EAM system uses catheter locations for anatomy surface creation [3]. Sciarra et al. evaluated CARTO^®^ 3 System ’s fast anatomical mapping (FAM) in 25 patients undergoing PVI [9]. FAM took $9\pm 3$ min, achieving an accuracy of $3.46\pm 0.02$ mm as validated by an MRI, with 96 percent vein isolation success. A recent method by Biosense Webster, “model FAM (mFam),” uses shape models and statistical modeling for LA reconstruction [10]. A blend of shapes models the atrium while relevant statistics of important anatomical features are modeled. This allows the generation of artificial LA shapes by sampling from the modeled distribution. The method shows promising results. However, its evaluation so far has been based on visual inspection by physicians and not quantitative measurements [11].

**Fig. 3:**
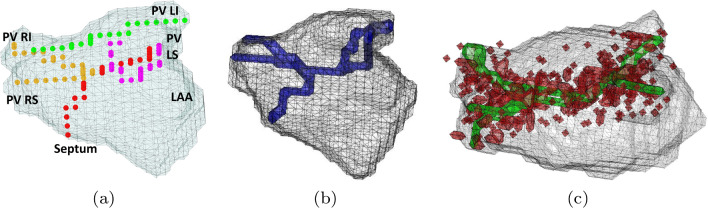
Synthetic path creation process. **a** Each color represents a traversal from one ostia to another. Septum to PVLS—red, PVLS to PVLI—purple, PVLI to PVRI—green, and PVRI to PVRS—brown. **b** A path composed of a concatenation of the traversals in (**a**). **c** The green composed path was augmented using random sampling around it to produce the red points

Works can be found in the literature that show success in reconstructing 3D shapes from partial data using generative networks as surveyed in [12]. The V-Net [13] is one of the possible generative networks. The network is a 3D voxel-based hierarchical multi-resolution convolutional network. Every resolution level includes several convolutions followed by downsampling for the next level. Once the lowest level is reached, the information is upscaled in levels using a “deconvolution” operation. This information is concatenated with the output of the same resolution level in the input as a residual input, which is convolved together. The output is a volume in the dimensions of the original input. In [14], a V-Net variant was used to reconstruct the LA shape using point clouds acquired by clinical mapping systems. In that work, 20–40% of the atria surface was sampled, to produce the point cloud sample input to the network. Strong dice scores and surface-to-surface distance were presented. In the current work, we are approaching the task of the LA shape reconstruction from a different perspective: we focus on a rapidly acquired catheter path that traverses anatomical landmarks only. Most of this path is in the blood pool, and we do not require it to touch any portion of the surface except the ostia of the PVs.

## Methods

This section describes our framework, which generates realistic sparse data from LA shapes and completes the full shape using neural networks. The system trains a learning algorithm to reconstruct the left atrium shape from catheter paths. Due to limited real data, we used simulated data with Biosense LA instance generator [10] and a catheter path generator. Figure 2 (left) illustrates data generation, creating LA samples with synthetic catheter paths. The network takes catheter trajectories to reconstruct LA surfaces, see Fig. 2 (right). Training involves generating path volumes resembling real scenarios from landmarks. In clinical cases, acquired catheter point clouds become voxel volumes, indicating catheter traversal. We show atrium instance sampling in “Input data generation: synthetic atria” section and graph-based path generation in “Input data generation: generating synthetic paths” section. Reconstruction using The proposed dense encoder–decoder (DED) is described in Section [3.3 and using V-Net in “Left atrial shape reconstruction using V-Net network” section.

### Input data generation: synthetic atria

We represent shape data as a $N=45^3$ voxel binary 3D volume ($2.666\,{\text {mm}}^3$ per voxel). Each voxel has the value of “1” for interior/boundary, and “0” otherwise-an occupancy volume. Using a predefined model [10], the atrium shape model blends parametric tube-like shapes transformed nonlinearly. Statistical modeling involves features like PV positions, orientations, and ridge locations as a multivariate normal distribution (MVN), learned from CT scans. A generated sample’s statistical score gauges its atrial representation likelihood within the model. To generate a synthetic left atria we sampled from the MVN of the model parameters. Then, we kept samples that scored well within the statistical model. Our resultant generated dataset contains 5006 and 1800 samples for training and testing, respectively.

### Input data generation: generating synthetic paths

The algorithm generates paths within LA shapes, emulating clinical catheter movement. With a single mapping sensor catheter, the path starts at the septum and traverses PVs sequentially: left superior, left inferior, right inferior, and right superior–distinct path sections. Path sections are depicted in Fig. 3a, and the full path is shown in b. Our vast LA dataset allows simulating diverse paths.

The process: We locate entry points at each PV ostium and then determine paths between ostia in a predefined order via graph optimization. This balances shortest distance and navigability, solved using Dijkstra’s algorithm [15]. We give an overview of the two main steps. More detailed steps are described in Supplementary material.

#### Simulated paths creation procedure overview

This algorithm processes input LA shapes as triangular meshes, with an initial mean shape derived from training data. Landmark points on this mean shape, including ostia and septal points, guide new input pose. Voxel-based representation is achieved by grid sampling within or over the mesh surface. Trajectories, originating at the septum and spanning through PVs’ ostia points, are defined by adjacent voxels. These paths usually curve toward the atrium center before reaching the next target. The algorithm then generates a volume where marked voxels indicate the path, while others remain zero.

#### Synthetic path augmentation

The generated synthetic path is next augmented by adding nearby points which are mostly inside the corresponding atrium. The augmentation procedure is motivated by the catheter’s physical setting and was tested empirically. The catheter vibrates during movement and may exceed the chamber boundary by slightly pushing it. First, for each (grid sampled) path point $\mathbf {x_p}$ we sample *n* points normally distributed around it, $\mathbf {x_n}\sim N(\mathbf {x_p},\sigma )$. Then the points are trimmed using a probability factor $s_{\textrm{f}}$, for each point. Next, we only consider points that are interior to the ground truth mesh. These undergo a normally distributed translation $\mathbf {t_{\textrm{f}}} \sim N(0,1)\cdot \mu _s$, where $\mu _s$ is the factor that determines the noise level. Figure 3c shows an augmentation result.

### Left atrial shape reconstruction using DED network

Next, we propose a reconstruction solution based on an NN recovery of complete LA shapes from sparse catheter paths, which we take as point clouds. We train a network on the generated synthetic paths, producing a probability volume via a final-layer sigmoid function. This signifies voxel likelihood of atrial interior/boundary. A 0.5 threshold binarizes values. We employ the dense encoder–decoder DED model, a multi-layer perceptron with input/output layers as linear voxel arrays. Interim layers are fixed size. Our model uses tied weights (only for input/output), masked input (akin to dropout after input), enhancing outcomes significantly. The Adam optimizer [16] is used. All layers, excluding the last, use RELU activation; the last employs sigmoid. Batch normalization [17] after each layer (but last) stabilizes activations, bridging synthetic clinical gap. The network combines binary cross-entropy loss (BCE) [18] and negative DICE coefficient [13] via linear mix. That is, for a ground truth volume *z* and a prediction *x*, the loss is

1 $$\begin{aligned} L(x,z) = \alpha {\text {BCE}}(x,z) - (1 -\alpha ) {\text {DICE}}(x,z). \end{aligned}$$

#### Spatial weight smoothing regularization (SWR)

In order to reconstruct a realistic atrial volume, we added a *Spatial Weight Smoothing Regularization (SWR)* term to the loss function. The loss including SWR is defined as:

2 $$\begin{aligned} L(x,z) + \lambda \sum _{j=1}^k \sum _{i=1}^N\left\| \nabla _{ \textbf{v} } {\textbf {W}}^{(j)}_i \right\| ^2 \end{aligned}$$

where *N* is the number of voxels, *k* is the hidden layer size, ${\textbf {W}}_{N\times k}$ denotes the layer weights. The SWR loss term is applied to the weights of the input and output layers only, for which each weight corresponds to a voxel. Specifically, each vector $\textbf{W}(j) = {\textbf {W}}_i^{(j)}$ assigns a weight for each voxel in the input volume if we flatten the volume to a vector. The gradient vector $\nabla _{ \textbf{v}}$ is computed for each element (voxel) in the $\textbf{W}(j)$ vector based on the original spatial locations of the volume. The spatial dimension is $\textbf{v}$, that is, the position within the volume for the three spatial axes, $\textbf{v}\in ({\hat{i}},{\hat{j}},{\hat{k}})$ in which the input and output reside. $\lambda$ represents the level of regularization.

The spatial derivatives are computed using finite differences. Figure S1 depicts the relationship between the voxels in space and the neurons in the layer and shows which weight difference is added to the cost.

#### Boundary enhancement mask

The most significant area in the volume is the surface boundary. In order for the model to have larger loss gradients around the surface boundary, we used a weighted DICE cost with a weighting mask $\Omega$. The mask assigns a weight for each voxel such that the majority falls over the boundary, and decreases for voxels further away from it, as seen in Figure S2. The weight of a voxel *v* is given by

3 $$\begin{aligned} \Omega (v) = (1+ \beta )/(1+PN(D(v))), \end{aligned}$$

where *D*(*v*) is the distance from the shape boundary and *PN* is the probability density function of the normal distribution with zero mean and $\sigma = 1.5$. The $\beta$ parameter was experimentally set to 14.

#### DED parameter selection

Following empirical experimentation, we report the results of the best-performing DED variant. To select parameters, we explored different scales; we then performed finer sampling around values that performed well over the test set. In our earlier work [8], we tested different combinations of depths and widths of the network architecture. The chosen variant includes two hidden layers with 350 neurons each. The cross-entropy and (weighted) DICE were combined in the loss function using $\alpha =\frac{2}{5}$. The variants are named according to the SWR parameter $\lambda$: “No SWR” with $\lambda =0$, “SWR005” $\lambda =0.05$, “SWR75” $\lambda =75$. “No Aug” net has the same parameters as “SWR005” but with no path augmentation and no boundary enhancement mask.

**Fig. 4:**
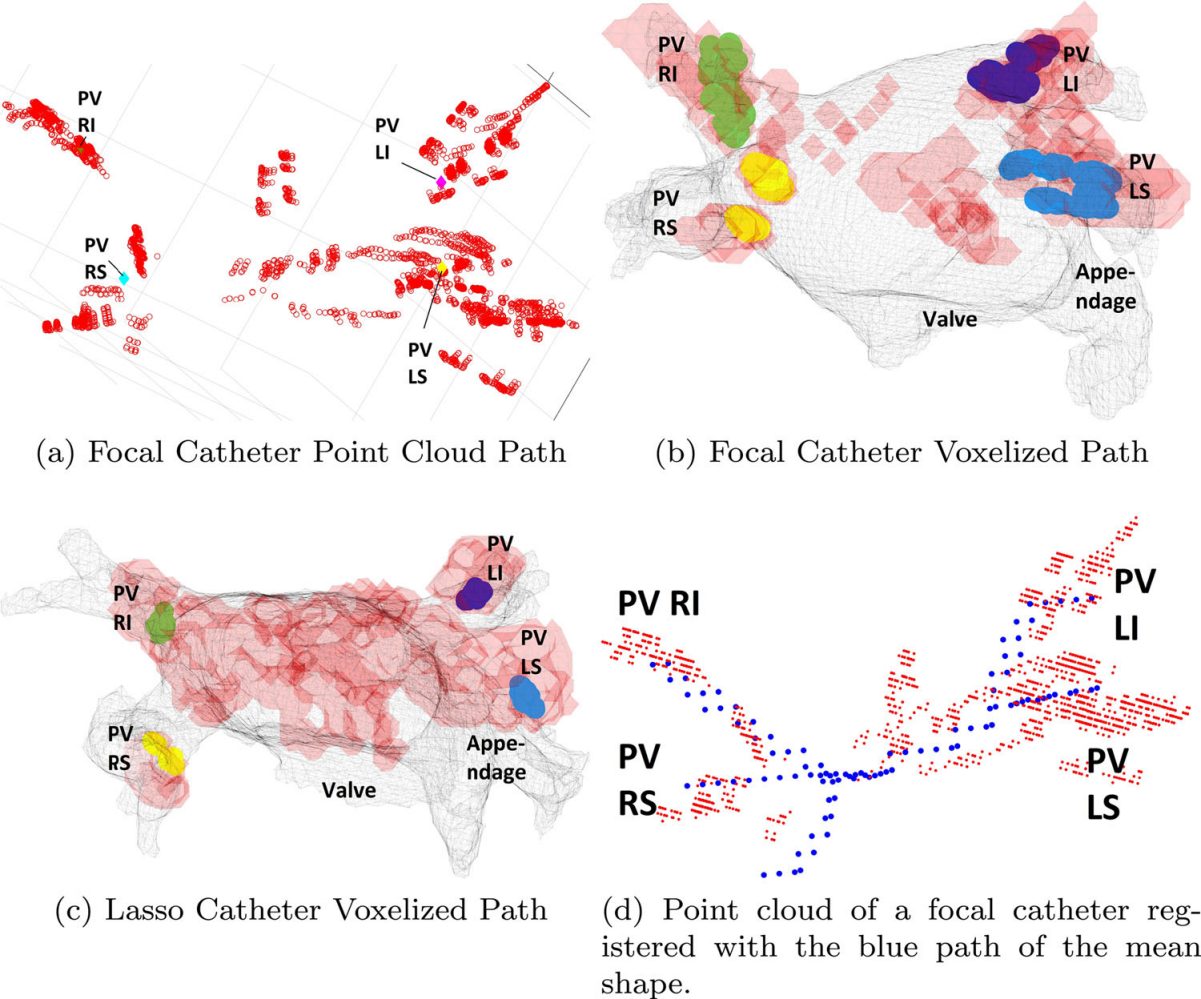
Input examples for the clinical cases. The acquired path is in red, the synthetic template path is in blue, and the CT is in gray. Tagged PV points are color coded

### Left atrial shape reconstruction using V-Net network

The V-Net [13] is composed of convolution layers followed by max-pooling, repeated over four stages to halve volume size. Subsequent steps involve four-stage up-sampling using learned filters, with residual connections concatenating input and earlier stage info of same size. Regularization involves batch normalization and dropout.

Figure 2 shows the architecture integrated with our system. Like the DED, the input is the voxelized catheter path and the output is LA reconstruction as occupancy volumes. Network parameters include filter count, down/upsampling layers, and concatenation. Notably, larger receptive field filters in the first and last layers enhance network performance.

### Mean shape as a baseline solution

To determine the effectiveness of any reconstruction algorithm, it needs to be compared to a common solution. In our case, we compared our results to the results of a mean shape solution. The mean shape was generated by taking a voxel-wise average over all the ground truth shapes in the training set. The mean location of a PV ostia over all the available atria data should converge to that of the mean shape. We defined a base coordinate frame for the reconstruction using the mean shape four PV ostia points. The input paths from the clinical cases were registered and transformed to have their PVs ostia match that of the mean shape (in the least squares sense) using rigid point set registration [19].

## Experiments and results

We next report the experiments and results in the reconstruction of the left atrial shape using experiments in human clinical cases. For this evaluation, we chose the networks with the best-performing parameter sets over the test set.

### Data acquisition and network input generation

Our clinical dataset was comprised of 80 cases with diverse catheters. Among these, 26 cases possessed CT-segmented registered meshes. Initial septum-based catheter paths connecting the PVs were obtained within 3 min during procedures, with physician-labeled PV ostia. Refer to Fig. 4a for the input point cloud (path) with tagged PV centroids. Figure 4b and c shows red input volumes with colored tagged points for each PV (yellow for PVRS, green for PVRI, blue for PVLI, light blue for PVLS) alongside registered CT.

The clinic-performed path deviates significantly from synthetic paths due to varied factors, including catheter velocity-related discontinuities and protocol deviations. Catheter type, maneuvers, and use of different catheters also contribute. We preprocess input for the network as follows. Coordinate systems alignment is performed between the acquired path and the network’s (mean shape-based, see “Mean shape as a baseline solution” section) coordinate systems. Mean PV ostia are derived from tagged points. Rigid point set registration [19] aligns acquired PV ostia with mean shape’s PV ostia (Fig. 4d). Transformation is applied to the acquired cloud. The transformed cloud is converted to an occupancy volume in $45^3$ voxels ($2.666\,{\text {mm}}^3$ each) representing point presence. This volume serves as network input, varying with catheter type (e.g., focal in Fig. 4b, round LASSO ^®^Catheter in Fig. 4c). The network output becomes a triangular mesh via Marching Cubes [20] algorithm in MATLAB, defined at 0.5 of output volume.

**Table 1:** Surface-to-surface distances (mean and standard deviation) comparing LA reconstructions to ground truth CT over 26 clinical cases

Distance from interest points	Unbounded	10 mm	15 mm	20 mm	25 mm	Hausdorff
*DED 0.05 SWR*						
Mean	6.057	**4**.**307**	4.697	5.083	5.363	24.292
Std	0.858	0.888	0.832	0.868	0.921	
*p* value (<)	0.055	*0.00054*	*0.00039*	*0.00058*	*0.0023*	
*DED 75 SWR*						
Mean	6.149	4.327	**4**.**678**	5.093	5.357	**23**.**418**
Std	0.979	0.803	0.712	0.767	0.837	
*p* value (<)	0.24	*0.0091*	*0.0045*	*0.009*	*0.016*	
*VNET*						
Mean	**5**.**962**	4.441	**4**.**678**	**4**.**984**	**5**.**216**	23.673
Std	0.913	0.75	0.708	0.651	0.662	
*p* value (<)	0.068	0.14	*0.045*	*0.025*	*0.022*	
*Mean atrium*						
Mean	6.241	4.735	5.103	5.458	5.686	23.934
Std	1.225	1.311	1.195	1.19	1.244	

Results are shown for DED, V-Net, and mean shape, in four different radii, and unbounded distance. The *p* value tests for significant differences between the network and the mean shape. *p* value under the significance level of 0.05 is in italics. In the rightmost column, we include a result of Hausdorff distance. Here we list the mean (over all cases) of the maximal error (mm). The bold is the best performing result for each test

### Evaluation with ground truth CT

We collected 26 properly registered CT cases, aligning acquired point cloud and network coordinate systems, enabled by CT. These cases, from three centers with even gender distribution, facilitated comparison between CT surfaces and reconstructed meshes using surface-to-surface distances. Focusing on clinically relevant LA regions due to surface area impact, we calculated distances within radii of physician-tagged points. The evaluation method involved finding nearest vertices on both meshes and averaging distances for symmetry. Only vertices within defined radii of tagged PV points were considered, where 15 mm radius captured PV ostia vicinity effectively (Figure S3).

Quantitative results for all 26 CT cases are shown in Table 1. We compare the surface-to-surface distances for “SWR005,” “SWR75,” V-Net, and the mean shape in various radii. The reported *p* value indicates the significance of improvement vs. the mean shape (paired *t*-test, one-tailed). Quantitatively, “SWR75,” “SWR005,” and V-Net decreased the distance error by 0.3–0.45 mm (statistically significant) over the mean shape results for radii between 15 and 25 mm. The mean Hausdorff distance [21], for all cases, is shown in the rightmost column.

Qualitative outcomes are demonstrated in the following figures.

**Fig. 5:**
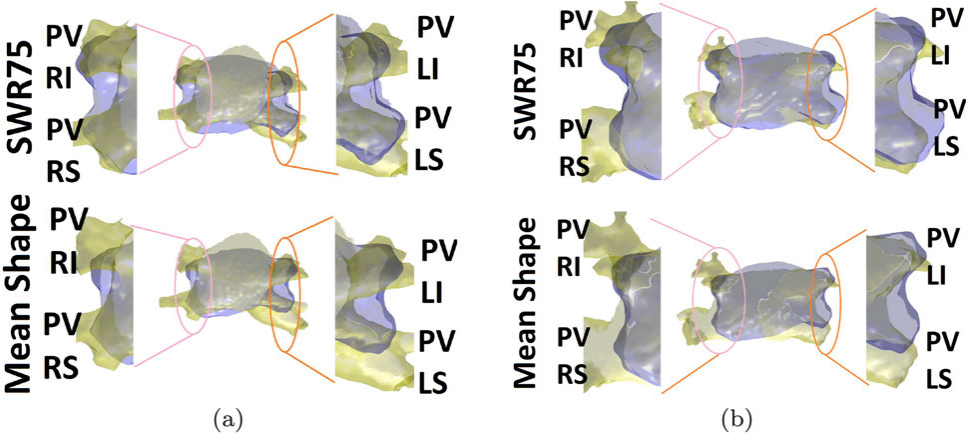
Comparison of reconstruction results focusing on the location and orientation of the PVs, for 2 sample cases: SWR75 reconstruction (top) mean shape reconstruction (bottom). CT ground truth is shown in yellow and the reconstruction results in blue. The visualization is clipped such that only the PV areas are shown. We note the better overlap of the blue and yellow regions in the SWR case

Figure 5 juxtaposes DEDand mean shape reconstructions, focusing on PVs, for two cases. CT ground truth in yellow, reconstruction in blue. Notably, DED enhances PVs’ location and orientation, ameliorating PVLS and PVRI discrepancies.

**Fig. 6:**
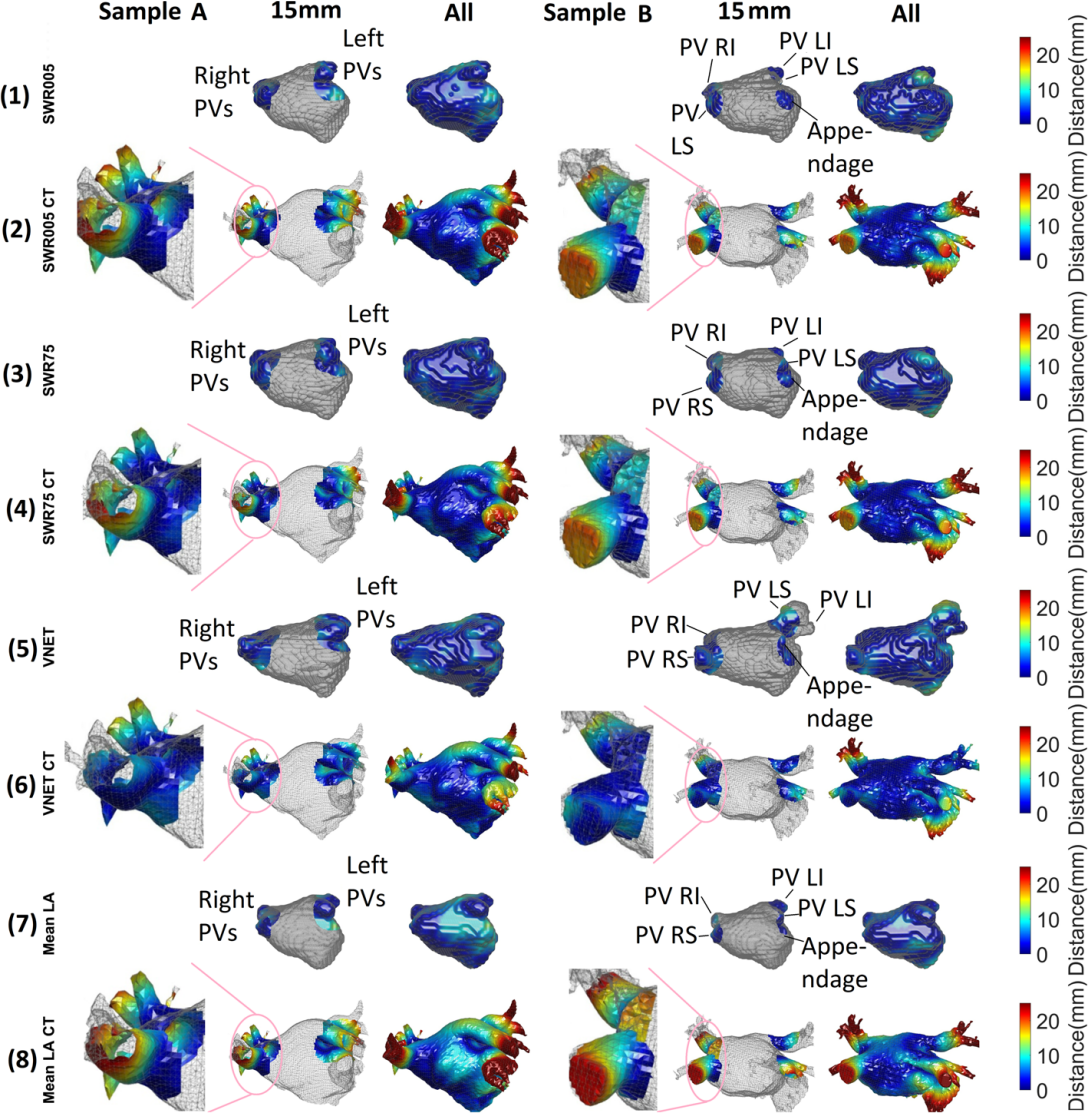
Visualization of the reconstructions in the clinical CT cases for the SWR005, SWR75, V-Net , and the mean shape. The surface-to-surface errors between the reconstruction and the CT, for 15 mm radius and unbounded radius, are shown. Each row is a similar view, where the first shows the reconstructions along with distances to the CT, while the second is the CT with distances to the reconstruction. Error with reconstructed PVs mostly affects the CT to reconstruction distance, and relevant sections are zoomed in. By comparing these regions, we see the improvement in different reconstructions over the mean shape solution

Figure 6 compares 4 methods (2 DED, V-Net, mean shape)—showing reconstructions and surface-to-surface distances. Odd-numbered rows depict reconstructions; even rows show CT surfaces. Distance maps within 15 mm of PV ostia and unbounded are displayed (as in Table 1). Odd rows indicate reconstruction-to-CT distances, even rows reverse. We note that errors are more pronounced in mean shape reconstruction—less dark blue (lighter scale). Right PVs are magnified, aiding visualization of differences.

**Fig. 7:**
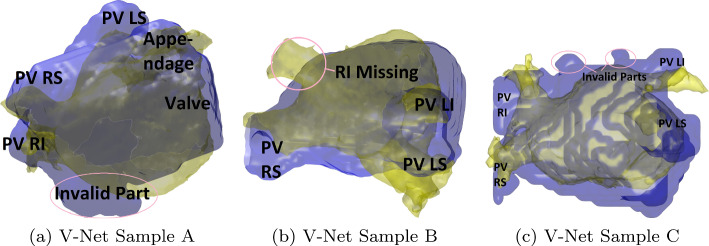
Invalid anatomy in V-Net reconstructions. The CT is in yellow, reconstruction is in blue, invalid anatomy is circled in pink

In some cases, V-Net produces smooth, low-error reconstructions. However, anatomical issues arise upon closer clinical scrutiny. Figure 7 illustrates such cases, where anatomical discrepancies appear, casting doubt on V-Net’s reliability.

**Fig. 8:**
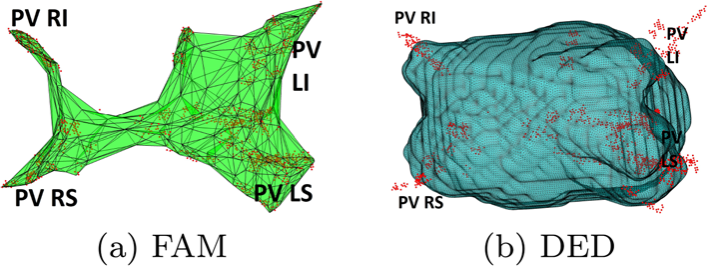
Comparing FAM and DEDreconstructions using the path of the initial bearing. Note that an anatomically correct solution is obtained only for DED

Figure 8 contrasts FAM and DEDreconstructions after the 3-min path acquisition. It is seen that FAM’s reconstruction lacks anatomical viability, while DEDremains anatomically accurate.

The DED network performs in real time. Using moderate consumer-grade hardware (Nvidia GTX2080), the run time for our DED networks with two hidden layers was less than 0.9 ms for 20 samples.

## Discussion and conclusion

This study employs a neural network-based strategy to reconstruct the left atrium shape from catheter paths. An anatomical model generates atrium shapes and an innovative algorithm constructs paths within them, serving as training data. Our findings highlight the efficacy of neural networks in reconstructing LA shapes from sparse point cloud paths.

Despite V-Net’s favorable quantitative metrics, its reconstructions often lacked anatomical fidelity, rendering it unsuitable for clinical use. The DED network, augmented with spatial weight smoothing and boundary enhancement, emerged as the best-performing option. It learned smooth, plausible atrium portions, culminating in realistic LA shapes.

Clinical cases were pivotal in assessing network performance. The DED solution exhibited consistency across various SWR parameter values, yielding statistically significant error reduction over mean shape for clinically relevant regions (CT evaluation). Emphasizing these areas is crucial, given that global metrics like surface-to-surface distance encompass numerous points susceptible to registration and motion errors. Networks fitting well over such data might improve global metrics while yielding flawed anatomy.

The balance between data fitting and anatomical fidelity is inherent. We favor networks that don’t overfit data, even when dealing with less similar inputs like clinical cases. DED without SWR introduced uncertainty and non-anatomical structures to accommodate dissimilar paths.

Qualitative assessment exposed V-Net’s non-anatomical outcomes, likely due to its local convolution approach. This outcome is consistent with the findings in [14] in which the authors show that their V-Net-based solution performance depends on a large percent coverage of the atria surface (note that this requires a longer acquisition time, as in FAM).

Our DEDsolution combines path acquisition (approx. 3 min) and real-time inference, enabling anatomically correct visualization within a third of FAM’s acquisition time. This aids procedures, novel workflows, and operator ease.

Our work seeks to create a system inferring anatomically plausible reconstruction while leveraging available data. Clinic-acquired data might offer insights such as force measurements and electrocardiograms, enhancing mapping. The network accommodates the widespread four PV anatomy and size variations; customized datasets should handle anatomical variations. This approach extends beyond LA reconstruction, being adaptable to other heart chambers, organs, imaging techniques, and partial inputs.

## Supplementary Information

Below is the link to the electronic supplementary material.

Supplementary file 1 (pdf 1686 KB)
