# Supplementary material for: Neural network reconstruction of the left atrium using sparse catheter paths
Source: Int J Comput Assist Radiol Surg. 2024 Sep 16;20(2):405–14. doi: 10.1007/s11548-024-03268-y (PMC11807916; doi:10.1007/s11548-024-03268-y)
Supplement: Supplementary file 1 — (pdf 1686 KB) [file 11548_2024_3268_MOESM1_ESM.pdf]

Supplementary Material

1 Supplementary Figures

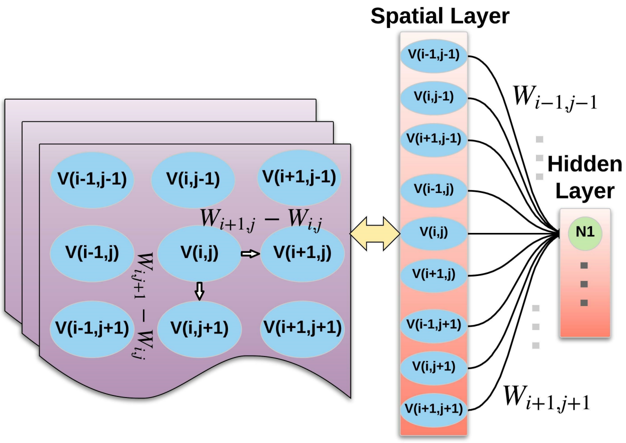

Fig. S1: Mapping the volume to the first and last layers. The left part shows a 3 by 3 neighborhood in a volume slice and to the right, it's mapping in the network. $V_{i,j}$ represents the voxel in row i and column j in the slice. $W_{i,j}$ is the weight between the spatial layer voxel $V_{i,j}$ and a neuron in the hidden layer. The white arrows indicate the weight derivative between neighbors that is added to the network cost. The slices of the volume are stacked.

This section presents the details of the synthetic path creation algorithm.

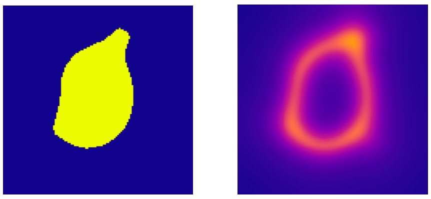

(a) (b)

Fig. S2: Boundary enhancement mask visualization. (a) A slice through the LA Volume, occupied space is in yellow. (b) Corresponding Weight map (arbitrary units), enhancing the LA boundary. High values are indicated by orange.

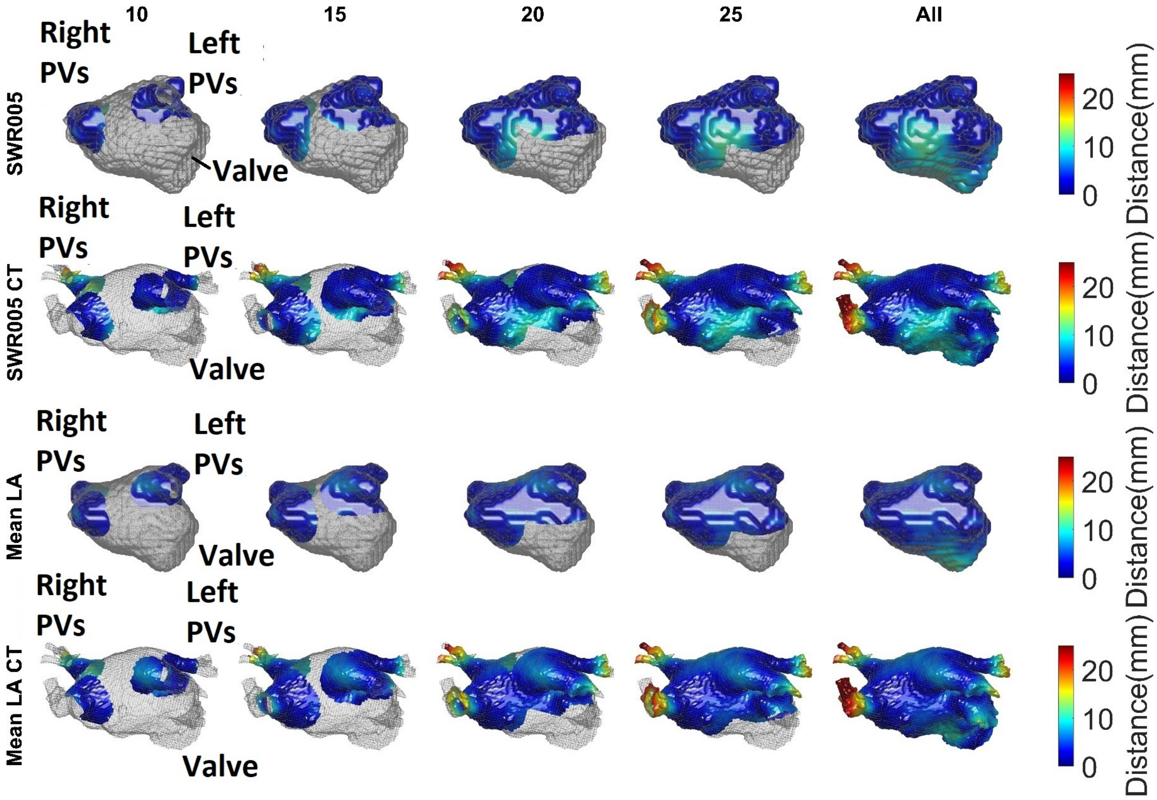

Fig. S3: Comparison of SWR005 and mean shape surface to surface distance for several radii (mm). Each row depicts the same view with different radii, while 'All' stands for unbounded radius. The first row for each model shows the reconstruction to CT distances while the second row is the CT to reconstruction distances.

2 Detailed Description of Synthetic Path Generation

2.1 Marking points in the PV's interior for all atria

In this stage, for each input atrium, we found a point for every PV that is inside the ostium. We utilized the mean LA shape reference, having manually selected a point on each artificially closed PV. The corresponding points on all the other LA samples were then generated by taking advantage of the fact that all atrial surfaces are similarly produced by the LA instance generator. These samples have Gaussian-like distribution for the orientations and the locations of the PVs. This step operated over the triangulated mesh representation to utilize the geometric properties of the surface. In the mesh representation, the chosen landmark points of the mean shape are vertices of the mesh.

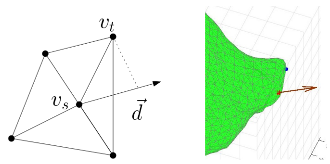

(a) (b)

Fig. S4: Illustration of local PV search. We choose vertices in the direction that matches PV orientation until no improvement can be made. (a) The direction $\vec{d}$ points from v_s to v_t as the next step. (b) The red Arrow shows the general orientation of the PV that should be followed.

Given the input atrium, we found for each landmark vertex (of the mean shape), the closest vertex over the input LA mesh. For each vertex, the normal (to the surface) at the abovementioned vertex of the mean atria is used as a direction to 'slide' across the new atrium vertices from the initial vertex until we can no longer advance in the direction of the normal, as seen in Figure S4. This vertex is likely to be on the same PV in the input atrium.

As shown in Algorithm 1 the input to the algorithm consists of the triangulated mesh (vertices and triplets of face membership) of the new atrium, a vertex p which is the original vertex in the mean atrium, a direction $\vec{d}$ which is the normal at p in the mean atrium and a threshold ϵ. In line 2 we assign the nearest vertex on the new atrium to the initial vertex from the mean atrium. Lines 4 - 6 compute the direction vectors from the current vertex to its neighbors. In Lines 7 - 9 we assign the neighboring vertex whose direction vector projection over $\vec{d}$ is maximal. The loop terminates once the projected step length is less than ϵ.

1 FindPointInPV $(mesh, p, \vec{d}, \epsilon)$
2 $current\_vertex \leftarrow FindNearestVertex(mesh, \text{p})$
3 **do**
4 $\quad neighbouring\_vertices \leftarrow getNeighbours(mesh, current\_vertex)$
5 $\quad \overrightarrow{dist\_vectors} \leftarrow neighbouring\_vertices - current\_vertex$
6 $\quad \overrightarrow{dist\_vectors} \leftarrow \frac{dist\_vectors}{\|dist\_vectors\|}$
7 $\quad direction\_difference \leftarrow \max \overrightarrow{dist\_vectors} \cdot \vec{d}$
8 $\quad current\_vertex \leftarrow arg\max_{v \in neighbouring\_vertices} \overrightarrow{dist\_vectors} \cdot \vec{d}$
9 **while** $direction\_difference \geq \epsilon$;

Algorithm 1: Find PV Algorithm

The septum point is a bit different to locate as it is chosen clinically by the physician on the septal wall between the right and the left atria. We decided to simulate this by finding the nearest vertex to a chosen septum in the mean

shape and then sampled a vertex (using a Gaussian distribution) around this vertex.

2.2 Creating a Path between two PV's

A catheter maneuver between two points inside the left atrium is very different from a straight line due to the limited degrees of freedom of the catheter. A realistic path between two PVs is relatively short and lies close to the center of the atrium (since a retraction to the center is usually performed while going from one area to the other). We express this problem by using a graph weighted such that the optimal path between two nodes will follow these two considerations. First, we use the discrete volume representation where voxels inside the atrium are represented by one. Next, we find the voxels that represent the two PVs. The graph is built such that each voxel in the volume is a node edge connected to its six neighbors. The boundary of the volume is extracted and a discrete signed distance transform (Euclidean distance) is computed over the volume. To compute this metric, a set of boundary voxels are selected having a distance of zero, and all other voxels are assigned a value that is the distance to the nearest boundary voxel. The value is negative for voxels outside the atrium, and positive inside. Thus, voxels close to the artium center have high Euclidean distance values. To define a cost for minimization, we denote the maximal distance as m_w and assign each voxel the weight $m_w - w_{dt}$ where w_{dt} is the voxel distance transform, as seen in Figure S5a. The edge between two neighboring voxels is assigned the mean of their weights. Edge cost increases as we move further away from the atrium's center. The shortest path between any two PVs is found using the Dijkstra algorithm [15], with regard to our objectives favoring a short path (since each edge adds weight) that is close to the atrial center. We also define an α parameter where each weight w becomes w^{α}. When α reaches zero the path tends toward the shortest path (as each weight becomes one), whereas α progresses towards one and beyond the path tends towards the center, see Figure S5b.

2.3 Path Part Integration

In order to create a full synthetic path we first we project the septum point from the template sample to the current sample. We then find point to point paths as described in Appendix 2.2. The first path is from the septum to the left superior, the second continues to left inferior, the third to the right inferior and the last one to right superior; see Figure 3a. Different parts of the path can be equilibrated by using a different power squashing (α) for each part, depending on how much we have to pull the catheter back to complete the path. The α parameter for each path was determined experimentally. The chosen values were $[0.001, 4, 1, 4]$ in the presented order. A sample path is shown in Figure 3b.

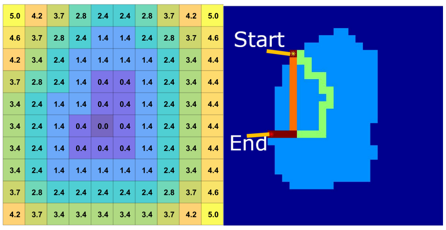

(a) (b)

Fig. S5: Path Weighting. (a) Cost of a vertex on the graph. The weight of the edge is the mean of its vertices. (b) α effect over the path. The orange path has $\alpha = 0$, and $\alpha = 1$ for the green path. The red area is common to both paths.
